# Influence of ZSM-5 Crystal Size on Methanol-to-Olefin (MTO) vs. Ethanol-to-Aromatics (ETA) Conversion

**DOI:** 10.3390/molecules28248046

**Published:** 2023-12-12

**Authors:** Daniel Dittmann, Elif Kaya, Dennis Strassheim, Michael Dyballa

**Affiliations:** Institute of Chemical Technology, University of Stuttgart, Pfaffenwaldring 55, 70569 Stuttgart, Germany

**Keywords:** ZSM-5 zeolite, methanol-to-olefin conversion (MTO), ethanol-to-aromatics conversion (ETA), solid-state NMR, acid sites

## Abstract

Crystal size is a key parameter of zeolites applied as catalysts. Herein, ZSM-5 crystals with similar physicochemical and acid properties, few defects, and aluminum exclusively in tetrahedral coordination are synthesized and the influence of the crystal size on the MTO and ETA conversion is investigated. Short olefins are the main products of the MTO conversion, whereas larger olefins and aromatics dominate the products after ETA conversion. In the case of both feeds, an increased crystal size decreases the catalyst’s lifetime. The MTO conversion over larger ZSM-5 altered the product distribution, which was not the case for the ETA conversion. The reason is that the instantly available aromatics during ETA conversion lead to fast coking and zeolite crystals only active in the outer layers. Thus, the different reactivity of different-sized ZSM-5 is direct proof of a different conversion mechanism for both alcohols.

## 1. Introduction

As an alternative production route to gasoline and olefins, the methanol-to-olefin (MTO) conversion route has gained frequent attention since the 70s [1,2,3]. Methanol is available in large scales from natural gas, for example, through stepwise selective oxidation [4,5,6]. The MTO reaction mechanism consists of two separate catalytic cycles [7,8,9], which is now referred to as a “dual-cycle concept” [10]. The larger alcohol homolog, ethanol, has the advantage of being considered “CO_2_-neutral” as it is generated in a large scale from biomass. This renders it a potential future platform chemical for the chemical industry [11,12,13]. In particular, ethanol is a promising feed for hydrocarbon fuel and aromatic formation via the ethanol-to-aromatics (ETA) conversion route [14,15,16,17,18]. The fundamental difference between methanol and ethanol feed is the present C-C bond in the latter case. This leads to different conversion mechanisms. In MTO conversion, surface methoxy groups (C_1_ species) are key intermediates for the generation of subsequent products [19]. In ETA conversion, oligomerization of ethylene (C_2_ species), formed instantly by dehydration, leads to the formation of larger hydrocarbons [15,20,21,22,23]. However, the present reaction types are comparable in both situations. Typical reaction types for both feeds are olefin oligomerization and cracking, hydrogen transfer, and aromatization, while methyl transfer and, further, hydrocarbon-pool reactions known from methanol conversion occur rather as side reactions for ethanol feeds [21,24]. In the past, the presence of similar intermediates was interpreted as if both feeds were interchangeable [20]. But since methanol was a cheaper feed, little research was conducted on comparing it with ethanol on similar catalysts. Especially the effect of the zeolite crystal size on the ETA conversion remains unclear, which is a fact that shall be changed with the present study.

A zeolite catalyst commonly applied for both the conversion of methanol and ethanol is the H-form ZSM-5 zeolite (structure type MFI), with crossing 10-membered ring (MR) pores (pore diameters of 0.51 nm × 0.55 nm and 0.53 nm × 0.56 nm). It is noteworthy that this zeolite structure can also catalyze the conversion of other biomass-derived oxygenate mixtures to hydrocarbons [25]. Both methanol and ethanol conversion are catalyzed by Brønsted acid sites (BAS). Thus, conversely, BAS properties significantly influence the product distribution that is received in conversions. Optimizing the catalysts’ BAS density was reported to lead to a selectivity to propylene over 50% [26]. Likewise, the selectivity of products can be derived from ethanol and regulated by adjusting the BAS density of the catalysts [15,22]. The importance of Lewis acid sites (LAS) for methanol conversion, on the other hand, was pointed out by Müller et al. [27]. LAS, associated with extra-framework cations, lead to hydrogen transfer and induce alkane and formaldehyde formation. This propagates the formation of aromatics if compared to a system with only BAS present. It was suggested that oxygenate species likewise lead to a faster deactivation when converting ethanol [15]. Thus, a comparable acidity is a prerequisite for comparing catalyst textual properties in MTO and ETA conversion routes. Note that some of the physicochemical properties of ZSM-5 zeolites tend to change drastically when different crystallite sizes are synthesized [28].

The influence of crystallite size on MTO conversion was frequently investigated, with often contradictive results when BAS density or other properties were ignored. Regarding unidimensional zeolite frameworks with only weak propagation of aromatic-based reaction cycles, a decreased crystal size leads, like desilication or milling, to an enhanced lifetime of the catalysts [29,30,31]. The reasons are shorter diffusion paths of coke precursors in zeolite micropores and thus slower coking. The impact of crystal size, even on lifetimes, is less clear if the literature on more complex 3D structures like MFI is considered. Most studies state that small crystal sizes lead to longer lifetimes during MTO conversion, but the literature is not uniform. In early investigations, ZSM-5 crystal sizes of 1–2 µm were beneficial for the formation of propylene and in particular ethylene [1,3]. Khare et al. [32] investigated multiple ZSM-5 catalysts with sizes between 2 nm and 17 µm and with *n*_Si_/*n*_Al_ ratios between 38 (largest crystals) and 87.5 (smallest crystals). It was concluded that the high residence time in larger ZSM-5 catalysts increased the propagation of the aromatic-based reaction mechanism, which in turn increases the selectivity to short olefins like ethylene in the conversion of dimethyl ether feeds. Especially studies that apply nanometer-sized ZSM-5 catalysts show frequently inconsistent results regarding the impact of crystallite size on product distribution and lifetimes [33,34,35,36]. Barbera et al. [37] resolved this issue by showing that the deactivation of such nanometer-sized ZSM-5 catalysts (100 to 300 nm, some desilicated) correlates primarily with the number of defects. In agreement with this work, it was recently shown that an amorphous shell can be found around nanometer-sized ZSM-5 catalysts. It decreases the amount of BAS and, potentially, also shape the selectivity effects of micropores on reactions [38]. Bleken et al. [39] investigated ZSM-5 catalysts in the range of ~100 nm to ~20 µm in the MTO conversion at 623 K and WHSV = 1.8 h^−1^. The BAS density determined by the H/D-exchange method varied from 0.26 to 1.00 mmol/g, but these results were inconsistent with the n-hexane cracking activity. The highest lifetime and selectivity to propylene were observed for large ZSM-5 crystals synthesized by the fluoride route. Also, Losch et al. [40] investigated high-silica ZSM-5 catalysts with 100 nm to 40 µm crystallite sizes in the methanol-to-propylene conversion (MTP) at 673 K and WHSV = 1.2 h^−1^. The BAS density varied, depending on the applied analysis method, in the range of 0.127 to 0.45 mmol/g. The BAS densities thereby exceed the maximum BAS density possible (0.13 mmol/g), which can be calculated from the *n*_Si_/*n*_Al_ ratio of the respective catalysts. The highest TOS of the catalysts was found for a catalyst with high BET (>400 m^2^/g), low BAS density (between 0.18 and 0.135 mmol/g), and “perfect” order. The authors attributed the significant TOS differences to the defect-free structure and the low acidity of a surprisingly large ZSM-5 catalyst with a crystal length of approximately 40 µm. Thus, in the latter two studies, very large crystals provided long lifetimes during MTO conversion. Also, Dai et al. [41] investigated SAPO-34 catalysts with a crystal size between 2.5 and 20 µm and with 1.00 to 1.25 mmol/g BAS. A faster deactivation occurred when larger SAPO-34 crystals were used. In summary, when it comes to the impact of crystal size, the literature on MTO is non-uniform, presumably because acid parameters and the number of defects were not comparable in many present studies.

For the conversion of ethanol, the crystal size remains a seldom investigated parameter. Meng et al. [42] investigated ZSM-5 zeolites with particle sizes between ca. 100 and 500 nm. All samples had similar BAS densities in the range of 0.32 to 0.27 mmol/g (presumed, unit not given). However, these values are impossible considering the *n*_Si_/*n*_Al_ ratio of ~70, which should result in 0.23 mmol/g BAS at maximum. Furthermore, the XRD patterns with only weak amorphous backgrounds are in harsh contrast to the reported average pore diameters [42]. These diameters, ranging from 2.5 to 5 nm, indicate the presence of mesopores in the investigated zeolites. However, these issues regarding physicochemical properties and acidity were not addressed by the authors. Again, it is noted that nanometer-sized ZSM-5 will usually contain an amorphous surface layer and improperly built-in aluminum [38]. Due to the high surface and many defects, the omnipresent Si(OH) groups will play a significant role in their deactivation [37]. This might explain why Meng et al. [42] reported more propylene and a longer lifetime when the smallest catalysts were used.

It is the aim of this study to clarify the size effect on the ETA conversion reaction and compare it with the effect on the MTO conversion. Therefore, the acidity of two ZSM-5 catalysts with different crystal sizes is investigated to exclude the influence of this parameter. Then, the samples are applied in MTO and ETA conversion. Differences observed when converting the two feeds are discussed, and the influence of crystal size on the reactivity is clarified for both feed molecules. This helps to identify differences between methanol and ethanol feeds and the influence of crystal size on the ETA conversion in particular.

## 2. Results and Discussion

### 2.1. Physicochemical Properties of the Catalysts

**Figure 1:**
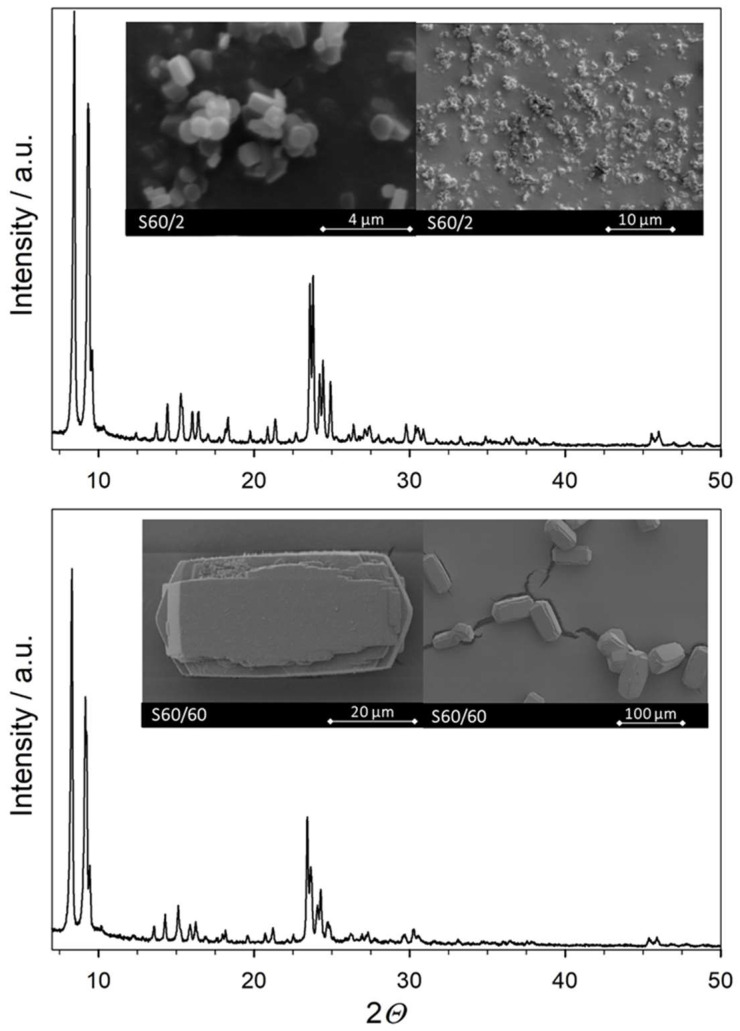
X-ray diffraction (XRD) powder patterns of investigated ZSM-5 zeolites and SEM images of catalysts in two magnifications.

**Figure 2:**
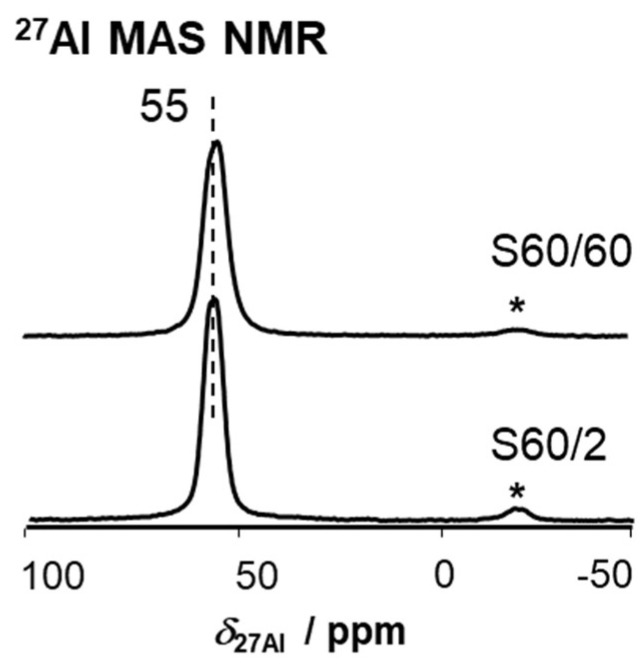
^27^Al MAS NMR spectra of both samples in a hydrated state. Spinning sidebands are indicated by asterisks (*).

First, the physicochemical catalyst properties are evaluated. ICP-OES measurements reveal *n*_Si_/*n*_Al_ ratios of 60 for both catalysts, with varied sizes of (maximum) 60 and 2 µm, respectively. The samples contained exclusively aluminum and silicon and were in fully exchanged H-form. This excludes the presence of transition metals that could potentially interfere with the reactions happening at the BAS. Representative SEM pictures of particles are shown in Figure 1. A low-resolution image, providing an impression of the agglomeration of the crystals, is shown on the left-hand side, while a high-resolution image of typical crystals is shown on the right. All crystals show the coffin-like shape typically observed for MFI crystals [43]. S60/2 crystals are sized 1–2 × 1 × 1 µm and only slightly agglomerated. No inhomogeneity associated with amorphicity is visible. For S60/60, large-sized crystals are found that are sufficiently big that they can even be investigated by applying optical microscopy. It is noted that such ZSM-5 crystals each consist of individual, intergrown structures [44]. Notably, such large crystals also show an inhomogeneous aluminum distribution, increasing from in- to outside [45]. X-ray powder diffraction patterns (XRD, see also Figure 1) of S60/2 and S60/60 show reflexes of a pure MFI phase without reflexes from competing phases. The relative crystallinity is 95% or above, which supports the impression from SEM pictures and confirms that only negligible amorphicity is present (see Table 1). The accessibility of the pore system and the accessible surface areas were investigated using N_2_-physisorption. The catalysts show a BET surface area of around 390 m^2^/g and similar micropore and total pore volumes as well as micropore areas. The N_2_-physisorption results thereby fit the typical literature values of ZSM-5 [15,26,46]. This clarifies that the pores of the catalysts are equally accessible. The next step in the characterization is the status of the framework aluminum. ^27^Al MAS NMR spectra contain a single peak at *δ*_27Al_ = 55 ppm for both S60/2 and S60/60 (see Figure 2). Note that aluminum, which gives rise to Lewis acidity, is usually found at a lower chemical shift after full hydration (saturation of the surface with water), for example, extra-framework aluminum at *δ*_27Al_ ≈ 0 ppm [47]. Thus, the ^27^Al MAS NMR spectra indicate the presence of exclusively tetrahedral framework aluminum in both samples S60/2 and S60/60 [15,26].

**Figure 3:**
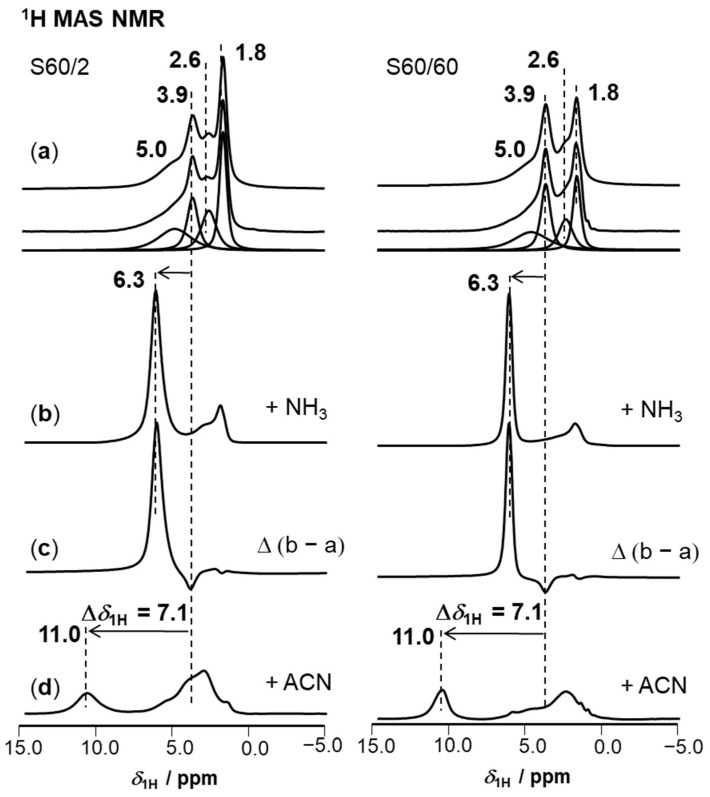
^1^H MAS NMR spectra of dehydrated catalysts. In line (**a**) (from top to bottom) the original spectrum, the total of the simulation and the deconvolution of the spectrum into four signals are found for each catalyst (see also Table 2). Below, the loadings with NH_3_ in line (**b**) and the difference spectra Δ(b − a) in line (**c**) are found. Spectra after loading with deuterated acetonitrile-*d*_3_ (ACN) are found in line (**d**). Intensities of spectra in lines (**b**,**c**) were reduced by ~25%.

Investigations by ^1^H MAS NMR spectroscopy, including de-convoluted spectra of dehydrated catalysts and spectra after probe loading, are shown in Figure 3, lines (a) to (d). A quantitative evaluation of the spectra is found in Table 2. The unloaded ^1^H MAS NMR spectra in Figure 3a consist of four individual peaks that can be assigned according to the literature [26,29,47]. The peak at *δ*_1H_ = 1.8 ppm corresponds to terminal Si(OH) groups. As no disturbed tetrahedral, pentahedral, or extra-framework aluminum is present, the presence of Al(OH) groups that contribute to a peak at *δ*_1H_ ≈ 2.6 ppm can be excluded. The peak is thus assigned to internal Si(OH) groups, whose increased chemical shift is caused by interaction with each other or the zeolite framework, respectively. Peaks at *δ*_1H_ = 3.9 ppm correspond to bridging Si(OH)Al groups, and broad peaks at *δ*_1H_ ≈ 5.0 ppm belong to disturbed Si(OH)Al groups that interact with other polar surface groups or the framework oxygens. Both types of Si(OH)Al groups can give rise to BAS density if they are accessible for molecules. In S60/60, a low amount of internal Si(OH) groups in a range around 0.1 mmol/g indicates that few defects exist, and only a slightly higher value of 0.18 mmol/g was determined for S60/2. In summary, the evaluation of ^1^H MAS NMR spectra showed rather similar properties of surface hydroxyl groups present on ZSM-5 catalysts. The next relevant parameter is the nature and quantitative amount of acidic surface hydroxyls in form of BAS that is present.

To determine whether all present Brønsted acid sites (BAS) contribute to the total acidity, the dehydrated catalysts were loaded with ammonia, and the peak caused by NH_4_^+^-ions was quantified. In Figure 3b, the respective ^1^H MAS NMR spectra of S60/2 and S60/60 are shown. The peaks at *δ*_1H_ = 3.9 ppm, assigned to acidic Si(OH)Al groups, and the peaks at *δ*_1H_ ≈ 5.0 ppm, assigned to disturbed Si(OH)Al groups, vanish. In parallel, new peaks at *δ*_1H_ = 6.3 ppm appear due to the NH_4_^+^-ions formed at the accessible BAS. Because of the high symmetry of these ions and the four-fold increased ^1^H MAS NMR intensity, this method enables an accurate determination of the BAS density [48]. Comparing values in Table 1 with deconvoluted ^1^H MAS NMR spectra in Table 2 supports the notion that all BAS are accessible for ammonia and thus also for potential reactants. Comparing the acid site density calculated theoretically from *n*_Si_/*n*_Al_ ratios (see Table 1), it is obvious that the BAS density of S60/60 is lower compared with that of S60/2. As there is no extra-framework aluminum present on the catalyst, the large crystals of S60/60 must contain inaccessible Si(OH)Al groups that may be located inside the crystal and in strong interaction with the framework, which leads to inaccessibility for NH_3_. Such hindered accessibility of a BAS is known in the literature and occurs often in small, inaccessible domains of zeolite material [49].

The exclusion of the presence of strong LAS is of further importance for our investigations. Herein, the amount of strong LAS was evaluated by quantifying adsorbed NH_3_ molecules according to the literature [50]. The respective difference spectra of ammonia loading and dehydrated samples are shown in Figure 3c. A negative peak at 3.9 ppm indicates the vanishing acidic proton of the bridging Si(OH)Al group upon reaction with ammonia to ammonium ions, NH_4_^+^. The NH_3_ coordinated at LAS appears as surplus ^1^H MAS NMR intensity below *δ*_1H_ = 3.8 ppm, with the peak intensity maximum around *δ*_1H_ ≈ 2.3 ppm. These peak areas account for <2% of the total ^1^H intensity after NH_3_-loading. Thus, the amount of strong LAS in our samples is negligible. A last question concerns the strength of the BAS in both catalysts. The adsorption-induced chemical shift Δ*δ*_1H_ of acidic protons, from *δ*_1H_ = 3.9 ppm to a lower field upon loading acetonitrile-*d*_3_ (ACN), is an established measure for the strength of BAS in zeolites [51]. The respective spectra after loading ACN are depicted in Figure 3d. It is noted that peaks caused by bridging Si(OH)Al groups show an adsorption-induced resonance shift to a lower field of Δ*δ*_1H_ = 7.1 ppm (error ± 0.1 ppm). This value agrees well with values reported for other high-silica ZSM-5 catalysts [15,26,46]. In summary, the density of strong LAS on the catalysts is negligible and the acid site strength of herein investigated catalysts is similar. This finding agrees with ^27^Al MAS NMR spectra indicating the exclusive presence of tetrahedral aluminum and with the chemical analysis that excludes the presence of impurities. It is concluded that the acid properties of S60/2 and S60/60 agree well.

### 2.2. Methanol-to-Olefin (MTO) and Ethanol-to-Aromatics (ETA) Conversion

**Figure 4:**
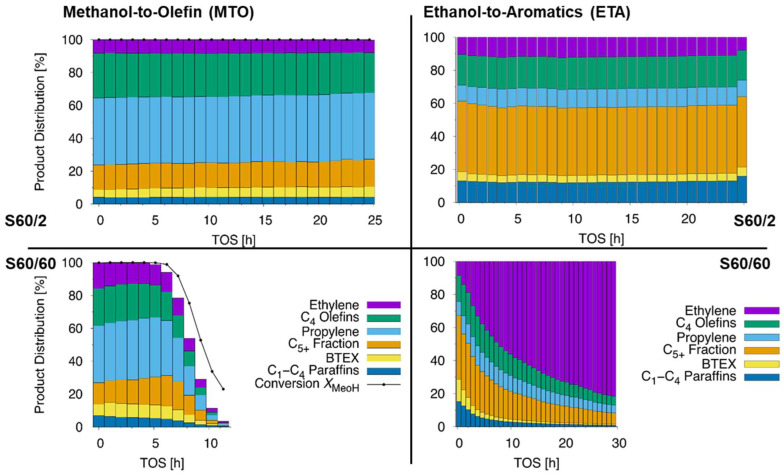
MTO conversion (**left**) recorded at 723 K and WHSV = 4 h^−1^, and ETA conversion (**right**) recorded at 673 K and WHSV = 1 h^−1^, both for ZSM-5 catalysts with (from **top** to **bottom**) increasing crystal size.

First, it is noted that comprehensive studies concerning the impact of process parameters on MTO and ETA conversion are available elsewhere [1,3,15,22]. The results of MTO conversion in a fixed-bed reactor at WHSV = 4 h^−1^ are shown in Figure 4, and relevant testing data, including typical descriptors for the dominating reaction cycle [46,52], are summarized in Table 3. Catalyst S60/60 is deactivated completely after 10 h, while catalyst S60/2 shows no sign of deactivation within 25 h TOS. This negative impact of large crystals on the lifetime is in line with most literature on MTO [26,30,31,41]. According to the literature, fewer pore entrances in larger crystals make pores easier to become clogged by coke, which in turn reduces the lifetime [44]. Both herein investigated catalysts had similar coke contents of 7% after full deactivation. The initial selectivity values gained over both catalysts, S60/2 and S60/60, are comparable. However, S60/60 clearly shows a higher selectivity to alkanes, ethylene, and BTEX aromatics (benzene, toluene, ethylbenzene, and xylenes), which are all products generated by the aromatic-based cycle of the MTO conversion. Upon beginning deactivation at WHSV = 4 h^−1^, a further increased selectivity to long-chain alkenes and a parallel decrease of propylene and butylene selectivity is observed for S60/60. This is in line with previous findings on ZSM-5 catalysts [26,49]. More products from aromatic-based cycles were previously explained by a longer residence time, which leads to a propagation of the respective cycles [32]. The hydrogen transfer index (HTI) was derived from the C_4_-fraction and is, with 0.07, lower for S60/2 than for S60/60, with 0.12. This again indicates a higher activity of aromatic reaction cycles for S60/60 [52]. An increased proportion of aromatic-based reactions over S60/60 is also supported by the lower C_3=_/C_3_ ratio [46]. One could argue here, that the presented ^1^H MAS NMR indicated a lower BAS density of S60/60 compared to S60/2 and that this could cause discrepancies. However, a decreased BAS density would lead to a higher C_3=_/C_3_ ratio, lower HTI index, and fewer aromatics and formed ethylene. This can be rationalized by comparing S60/2 with a catalyst of higher Si/Al ratio (130) from the literature [26]. For this pair, a more converse trend in product distribution changes and descriptors is observed than when comparing to the pair S60/60 and S60/2. Thus, the slightly different BAS density is not the reason for the observed selectivity change. It is thus concluded that in larger ZSM-5 crystals applied in the MTO conversion, the selectivity of ethylene, BTEX aromatics, and alkanes are increased at the expense of propylene due to propagation of aromatic-based reaction cycles in larger crystals of S60/60. Our conclusion agrees with the findings of Khare et al. [32] and with the higher propylene and lower BTEX selectivity reported due to a shortened residence time in plate-like ZSM-5 crystals [53]. It is noted that similar conclusions were drawn for differently sized SAPO-34 catalysts [41].

The corresponding ETA conversion results are shown in Figure 4 and Table 3. The feed ethanol is instantly dehydrated to ethylene, a reaction mechanistically separated from the subsequent oligomerization, aromatization, and cracking reactions [23]. Ethylene can thus be considered to be a product of the ETA conversion or the reactant. The authors stick to the latter, but this definition is not uniformly used in the present literature [15,22]. For clarity, no value for the conversion of ethanol will be given in this work. Similar to the MTO conversion, the smaller catalyst S60/2 shows a longer lifetime and no deactivation within 25 h. In contrast, S60/60 shows already initially unconverted ethylene, which equals an incomplete conversion, and a fast deactivation over the first hours. It can thus be clarified that, in agreement with previous findings [42], the primary effect of an increased crystal size is a decreased lifetime for ethanol conversion. This is reasonable, as the deactivation in both reactions is caused by coke formation and pore-clogging. The smaller amount of pore openings for large crystals enables a faster inactivation of pores by coke. The finally reached coke contents in the catalysts are comparable and lie between 7 and 8% of the total mass.

The monitored ETA conversion results are, however, different from those from MTO conversion if the observed selectivities are considered. We note the almost identical product distribution during ETA conversion. For S60/60, compared with S60/2, only a slightly increased ethylene and BTEX content, as a result of the fest deactivation, is observed. This is in contrast to the work of Meng et al. [42], who reported significantly more aromatics for larger ZSM-5 crystals. However, as discussed previously, their nano-sized catalysts had unclear physicochemical and acid properties that could have interfered with the crystal size. The results presented therein can, for example, be understood if we remember that the authors found mesopores on their catalysts. It was in that recently shown respect that mesoporosity can lead to an increased selectivity to aromatics in the ETA conversion [14]. When it comes to S60/60 in the ETA conversion, herein, the ratio between the different products is not significantly changed, the final coke contents are similar, and the calculated mechanistic descriptors, C_3=_/C_3_ ratio and HTI, of S60/2 and S60/60 are almost identical. This discrepancy between MTO and ETA conversion can be rationalized as both feeds are converted by different reaction mechanisms [21]. Larger crystal sizes support the aromatic-based reaction cycles in MTO conversion [32]. However, before aromatics are formed, olefins and alkanes are the predominant species. Thus, their aromatization requires a longer residence time [24]. In contrast, for the ETA conversion, herein, no differences in the selectivities are found. Especially the instant presence of unreacted ethylene during ETA conversion over S60/60 indicates a fast deactivation due to coking. This is reasonable, as during ETA conversion, mechanistically running over ethylene (C_2_-species) oligomerization and subsequent aromatization, aromatics are formed much faster than during MTO conversion (reactant is first a C_1_-species) [15,23]. Thus, instantly, aromatics are available for coking during ETA conversion, while during MTO conversion olefins are the predominant species. These aromatics lead to fast coking of the H-ZSM-5 catalyst. Supporting this, it was shown that coking with alkanes happens first inside and then outside of the pores, while the coke from aromatics is deposited on the outer parts of the crystal and decreases the diffusion of the intermediates [54]. Conclusively, in the ETA conversion, only the outer crystal parts of microporous ZSM-5 zeolites are active, while during MTO conversion, reactions inside inner pores occur to a higher extent. This leads to a longer residence time inside larger particles and a changed selectivity during MTO conversion. This explains why post-modifications that enhance the coke resistance of the ZSM-5 catalyst, like mesopore formation, are very promising strategies to increase the lifetime of ETA conversion catalysts [14,18]. Thus, it was herein clarified how one parameter, ZSM-5 crystal size, impacts the ETA product distribution. This makes the mechanistic differences between the two industrially important sister reactions, methanol and ethanol conversion, obvious.

## 3. Materials and Methods

### 3.1. Sample Preparation

Zeolites S60/2 were synthesized as described elsewhere [26]. Briefly, 21.8 g tetraethylorthosilicate (Merck, Darmstadt, Germany) was hydrolyzed by stirring in alkaline tetrapropyleammoniumhydroxide (TPAOH) water solution (3.5 g of 40% TPAOH solution from Chempur (Karlsruhe, Germany) diluted with 56.5 mL water) overnight. Subsequently, 10 mL water was added, and then a solution of 0.44 g aluminumisopropoxide, 0.16 g NaOH, and 3.3 g TPAOH in 50 mL water was added. The mixture was stirred for an additional 30 min, and the zeolite was crystallized over 26 h at 433 K in 120 mL stainless steel autoclaves with a Teflon inlet. The template was removed by calcining in flowing synthetic air at 823 K for 24 h. Zeolite S60/60 was synthesized according to Kornatowski [43]. Briefly, 7.05 g tetrapropyleammoniumbromide (Acros, Geel, Belgium), 5.98 g NaHCO_3_ (Aldrich, Steinheim, Germany), and 0.11 g Al(OH)_3_ (Merck) were diluted in 50 mL water. A solution of 40 g Ludox HS40^®^ (Aldrich) in 38 mL water was added. The mixture was stirred only 3 min prior to transfer in 120 mL stainless steel autoclaves and crystallized over 5 days at 843 K. The template was removed by calcining in flowing synthetic air at 847 K for 216 h. All zeolites were 3× ion-exchanged with 1 M NH_4_NO_3_ solution and washed nitrate-free prior to use.

### 3.2. Characterization Methods

Chemical analysis was performed using a Varian VISTA-MPX ICP-OES instrument. X-ray diffraction was measured using a Bruker D8 diffractometer with CuK*α* radiation (*λ* = 1.5418 Å) in a 2θ range of 5–50° applying a scan rate of 6.0°/min. The relative crystallinity was determined by comparing areas of crystal scattering and amorphous scattering using the Bruker software EVA (2.6.1). The chemical composition of the catalysts was obtained by an ICP-OES instrument IRIS Advantage. Physisorption with N_2_ was conducted on a Quantachrome Autosorb 3B instrument at 77 K. Before the measurement, the samples were outgassed for 16 h at 623 K. The catalyst nomenclature reflects the *n*_Si_/*n*_Al_ ratio determined by ICP followed by the size in µm. For example, S60/2 has a *n*_Si_/*n*_Al_ ratio of 60 and a crystal size of 2 µm. ^1^H and ^27^Al MAS NMR measurements were performed with samples in the dehydrated or hydrated state on a Bruker Avance III 400 WB spectrometer at a sample spinning frequency of 8 kHz (MAS). The two nuclei were measured at resonance frequencies of 400.1 and 104.3 MHz, upon π/2 and π/8 single pulse excitation, with repetition times of 20 s and 0.5 s, and by accumulating 80 and 4800 scans, respectively. The sample dehydration was carried out at 673 K in a vacuum (pressure below 10^−2^ Pa) for 12 h (heating ramp below 2 K/min). For quantitative ^1^H MAS NMR measurements, a dehydrated zeolite H,Na-Y (ammonium exchange degree of 35%) was used as an external intensity standard. The activated samples were treated in a glove box purged with dry N_2_. The samples were loaded with 70 mbar acetonitrile-*d*_3_ (99.9% deuterated, Acros) via a vacuum line and then evacuated at 293 K for 12 h before measurement. For determining the BAS density, the pre-activated samples were loaded with 60 mbar ammonia gas for 10 min over a vacuum line and subsequently evacuated at 453 K for 2 h. Full hydration was achieved by storing the samples 24 h over an aqueous, saturated Ca(NO_3_)_2_ solution. Spectra were simulated using the Bruker software WINNMR (6.2.0.0) and WINFIT (#961107).

### 3.3. Catalytic Testing

The catalytic conversion of methanol was investigated at atmospheric pressure in a fixed-bed glass reactor (inner diameter 5 mm) with 0.1 g of catalyst, sieve fraction 0.25–0.6 mm. These were filled into the fixed-bed reactor and activated under flowing nitrogen gas at 723 K for 4 h. At the reaction temperature of 723 K, methanol was introduced by saturating a N_2_ gas flow corresponding to the weight hourly space velocity of WHSV = 4.0 h^−1^. The reaction products were analyzed by on-line gas chromatography with an HP5890/II gas chromatograph equipped with a flame ionization detector (FID) and a 50 m capillary-column Poraplot Q (Agilent). After the reaction, the used catalysts were slowly cooled down to room temperature under N_2_, and the completely coked fraction was analyzed by TGA. For the ethanol conversion, comparable equipment was used as described previously [15]. A fixed-bed glass reactor (inner diameter 7 mm) was filled with 0.46 g of the catalyst (sieve fraction 0.2–0.32 mm) diluted by inert sea sand. The catalyst was activated under nitrogen flow at 723 K for 0.5 h. After cooling down to the reaction temperature of 673 K, ethanol was introduced by typically flowing 15 mL h^−1^ through a saturator filled with chromosorb and ethanol. This corresponds to a WHSV of 1.0 h^−1^. The deactivated catalysts were cooled down to room temperature under N_2_, and the completely coked fraction was analyzed by TGA.

## 4. Conclusions

In this study, two ZSM-5 catalysts with different crystal sizes but similar textual and acid properties were synthesized. Both samples showed a pure MFI phase, no amorphicity or defects, and comparably high surface areas. No extra-framework aluminum appeared in ^27^Al MAS NMR spectra and no associated Lewis acid sites (LAS) were found. Both the catalyst’s Brønsted acid site (BAS) density and its strength were investigated by probe molecules and found to be comparable. With these prerequisites, an influence of acid or physicochemical properties on the obtained product distributions could be ruled out.

In the MTO conversion, the ZSM-5 catalyst with smaller crystals results in a significantly enhanced lifetime. However, discrepancies in the product distribution of the MTO conversion between ZSM-5 of different sizes were observed. In particular, the conversion over larger S60/60 crystals resulted in more alkanes, ethylene, and aromatics. This was accompanied by a higher HTI index and a smaller C_3=_/C_3_ ratio. Both the changed selectivity and descriptors indicate a stronger propagation of aromatic-based reaction cycles, due to a longer residence time, when increasing the crystal size in the MTO conversion. Thus, the crystal size of ZSM-5 crystals has an impact on the proportions of MTO reaction cycles.

Also, in the ETA conversion over the ZSM-5 catalyst, smaller crystals result in a significantly enhanced lifetime. However, no influence of the crystal size on the ETA product distribution is observed. Also, the applied mechanistic descriptors like the HTI index and C_3=_/C_3_ ratio do not indicate mechanistic changes. The ETA conversion mechanism involves, in contrast to MTO conversion, a homologation sequence that leads much faster to aromatics, the coke precursors. Initially, deactivation of the catalysts due to coke is observed, especially on large ZSM-5 crystals. The pores are, obviously, clogged too fast by the instantly formed aromatics, which prevents longer residence times. Thus, the mechanistic differences between methanol and ethanol conversion are evidenced by the influence of crystal size on the respective product distributions. Thus, in the ETA conversion, in contrast to the MTO conversion, the crystal size can be changed without having to consider selectivity changes.

## Figures and Tables

**Table 1:** Physicochemical properties and acidity of ZSM-5 catalysts.

Catalyst	*n*_Si_/*n*_Al_ Ratio ^a^	Theoretical BAS Density [mmol/g] ^a^	Measured BAS Density [mmol/g] ^b^	Crystal Size ^c^ [µm]	Rel. Crystallinity ^d^ [%]	BET [m^2^/g]	Micropore Volume [mL/g]	Micropore Area [m^2^/g]	Total Pore Volume [mL/g]
S60/2	60	0.27	0.27	<2 × 1 × 1	99	387	0.13	244	0.21
S60/60	60	0.27	0.22	60 × 30 × 30	95	394	0.11	205	0.21

^a^ Calculated from ICP-OES, accuracy ±10%. ^b^ From ammonia loading via quantitative ^1^H MAS NMR spectroscopy, accuracy ±5%. ^c^ From SEM images. ^d^ From XRD patterns, accuracy ±5.

**Table 2:** Quantitative deconvolution of ^1^H MAS NMR spectra in Figure 3a. All values are in mmol/g with an accuracy of ±10%. The BAS density after NH_3_-loading is found in Table 1.

Catalyst	Disturbed Bridging Si(OH)Al Groups (~5.0 ppm)	Bridging Si(OH)Al Groups (3.9 ppm)	Internal Si(OH) Groups (2.6 ppm)	Si(OH) Groups (1.8 ppm)
S60/2	0.16	0.11	0.11	0.18
S60/60	0.11	0.11	0.07	0.10

**Table 3:** Selectivity and mechanistic descriptors of MTO (TOS = 3 h, WHSV = 4 h^−1^) and ETA conversion catalysts (TOS = 1 h, WHSV = 1 h^−1^).

Catalyst	*S*_AlkanesC1-C4_	*S*_Ethylene_	*S*_Propylene_	*S*_Butenes_	*S*_C5+_	*S*_B__TEX_	C_3=_/C_3_	HTI C_4_	Coke ^a^ [wt%]
Methanol-to-Olefin (MTO)									
S60/2	4	8	41	27	15	5	32	0.07	7
S60/60	6	13	36	22	15	8	24	0.12	7
Ethanol-to-Aromatics (ETA)									
S60/2	13	13	11	19	25	19	2.8	0.29	8
S60/60	13	14	11	19	23	20	2.8	0.31	7

^a^ Taken from the fully deactivated catalyst fraction.

## Data Availability

The data presented in this study are available on request from the corresponding author. The data are not publicly available as no supporting data exceeding this report was generated.
